# Immunoablation and Stem Cell Transplantation in Amyotrophic Lateral Sclerosis: The Ultimate Test for the Autoimmune Pathogenesis Hypothesis

**DOI:** 10.3389/fneur.2016.00012

**Published:** 2016-02-11

**Authors:** A. Arturo Leis, Mark A. Ross, Joseph L. Verheijde, Jose F. Leis

**Affiliations:** ^1^Department of Neurology, Mayo Clinic Arizona, Scottsdale, AZ, USA; ^2^Center for Neuroscience and Neurological Recovery, Methodist Rehabilitation Center, Jackson, MS, USA; ^3^Department of Physical Medicine and Rehabilitation, Mayo Clinic Arizona, Scottsdale, AZ, USA; ^4^Division of Hematology Oncology, Mayo Clinic Arizona, Phoenix, AZ, USA

**Keywords:** autoimmunity, amyotrophic lateral sclerosis, stem cell transplantation, immunosuppression

Amyotrophic lateral sclerosis (ALS) is a fatal neurodegenerative disorder characterized by a progressive death of motor neurons for which there is no cure or effective treatment. The cause of ALS and the specific mechanisms of neuronal death remain unknown. However, considerable evidence supports the existence of autoimmune mechanisms contributing to pathogenesis in ALS, including biochemical, morphological, pharmacological, and physiological studies performed in animal models, cell culture, or with *ex vivo* preparations (1–6). Typical hallmarks of autoimmunity, such as circulating immune complexes, higher frequency of a particular histocompatibility type, or association with other autoimmune diseases, have also been reported (7, 8). An important marker of autoimmunity is the degree of T-lymphocytic infiltration in the anterior horn of the spinal cord from ALS patients (9, 10). Using monoclonal antibodies against T-cells, B-cells, and macrophages, almost 80% of the specimens show a cellular mononuclear infiltration. The cellular composition of the spinal cord inflammation consists of subsets of suppressor or cytotoxic T-cells and macrophages in the anterior and lateral corticospinal tracts and anterior horns (10). T-helper cells are also observed in proximity to corticospinal tract degeneration (11). Hence, inflammation in ALS spinal cord and brain appears to be primarily due to T-cells and macrophages (12), and aberrant macrophage activity is believed by many investigators to contribute to the pathology underlying ALS. This may explain the recent promising results of an ALS phase 2 clinical trial of NP001, a regulator of inflammatory macrophage activity (13). Although the predefined endpoints in this study did not reach statistical significance, administration of NP001 was associated with cessation in disease progression in 27% of patients, approximately 2.5 times greater than the percentage in patients on placebo. Two major plasma markers of inflammation, interleukin-18 (IL-18) and lipopolysaccharide (LPS), differentiated NP001 responders from non-responders, suggesting that the subgroup of patients with greater baseline biomarkers of neuroinflammation experienced the most benefit (13). Additional evidence pointing toward pathologic involvement of autoimmune processes has been the finding that immunoglobulins from ALS patients have been shown to cause apoptosis of motor neurons in primary spinal cord cultures (14) and that passive transfer of immunoglobulins to mice caused abnormalities at motor end-plates and degeneration of motor neurons (4, 15). These findings suggest that antibodies can contribute to disease pathogenesis. Increased levels of interleukins IL-17 and IL-23 have also been found in serum and cerebrospinal fluid of ALS patients (16). This increment is thought to be a sign of T-helper 17 (Th17) activation, a subset of T-cells suggested to be crucial in destructive autoimmunity. Astrocytes have also been shown to participate in the pathogenesis of ALS by producing a microenvironment toxic to motor neurons through increased neuroinflammation, oxidative damage, and glutamate excitotoxicity (17, 18). Overactivated astroglia produce high levels of protein S100B and other proinflammatory factors, which exacerbate neuroinflammation. The extracellular effects of S100B vary, depending on the concentration attained; at nanomolar concentrations, S100B is trophic to neurons, but at micromolar concentrations, S100B causes neuronal apoptosis (19, 20). Many of the effects of S100B on neurons are transduced by the receptor for advanced glycation end-products (RAGE), which participates in the pathophysiology of brain inflammatory disorders by regulating several inflammation-related events, including activation and migration of microglia and neutrophils to inflammatory sites (19–21). Extravasation of S100B into the systemic circulation can also trigger a pathologic autoimmune reaction with circulating antibodies that may re-enter the CNS to initiate an autoimmune response (22). Hence, S100B can be viewed as an astrocytic endokine that can act as an immunoregulator to participate in inflammation and autoimmunity. Additional support for the autoimmune pathogenesis hypothesis is the finding that ALS has recently been included in the spectrum of neurologic manifestations associated with voltage-gated potassium channel (VGKC) autoimmunity (23–25).

Because of the large body of evidence suggesting a neurotoxic effect of the immune response in ALS, numerous therapeutic trials based on the autoimmune pathogenesis hypothesis have been performed. However, these studies have failed to demonstrate improvement in motor function. Immunosuppressive drugs, such as corticosteroids, azathioprine, cyclophosphamide, cyclosporine, or combination pharmacotherapy, as well as immunotherapy with plasmapheresis or intravenous immunoglobulins, have not altered disease progression (26–28). Moreover, in what had been considered the ultimate trial in immunosuppression for ALS, total lymphoid irradiation (TLI), which produces a more powerful and prolonged immunosuppression, did not benefit patients with ALS (29). The conclusion from these therapeutic trials was that autoimmune mechanisms did not contribute to the pathogenesis in ALS. However, all negative trials based on the autoimmune pathogenesis hypothesis were performed in the latter decades of the 1900s, and TLI and the immunosuppressive drugs used in these early trials are no longer considered today’s gold standard in immunosuppression.

Since 1996, intensive immunosuppression followed by autologous hematopoietic stem cell transplantation (AHSCT) to renew the immune system has been used for the treatment of severe autoimmune diseases refractory to approved therapies (30). The largest cohort studied worldwide (European Group for Blood and Marrow Transplantation registry between 1996 and 2007) evaluated the long-term outcomes of these transplants in 900 patients with various autoimmune diseases including multiple sclerosis, systemic sclerosis, systemic lupus erythematosus, rheumatoid arthritis, juvenile arthritis, and hematologic immune cytopenia (30). Among all patients, the 5-year survival was 85% and the progression-free survival 43%. Age <35 years and transplantation after the year 2000 were associated with progression-free survival. This worldwide study showed that profound immunosuppression and AHSCT can induce sustained remissions in patients with severe systemic autoimmune diseases refractory to conventional therapy (30). Furthermore, it underscored the therapeutic principle that in all successfully treated autoimmune diseases, there are intractable cases that subsequently improve with more aggressive immunotherapy. Hence, the failure of prior ALS therapeutic trials based on the autoimmune pathogenesis hypotheses may have reflected a relative inability to achieve the intense immunosuppression needed to abolish the immune mechanisms contributing to disease progression.

In recent years, it has been recognized that stem cells have the ability to provide numerous potential benefits in ALS (31) and a variety of stem cell-based therapies are underway (32). The majority of these studies have assessed the therapeutic efficacy of different cellular delivery mechanisms, including intracranial, motor cortex, intrathecal, intraventricular, intraspinal, and intravenous, utilizing various types of stem cells to renew, repair, or replace damaged motor neurons. However, perhaps because of the earlier negative trials that discredited the autoimmune pathogenesis hypothesis, these stem cell trials do not emphasize immunosuppression as a therapeutic strategy. Rather, the injection of various types of stem cells into brain or spinal cord for the purpose of replacement or repair of damaged neurons, without antecedent immunosuppression, is perceived as holding the greatest potential for eventual success in the ALS patient (33). The omission of immunosuppression as a therapeutic strategy is problematic, since copious scientific studies support a role for neurotoxic effects of the immune response in ALS. Even the pioneers who used TLI and other immunosuppressive agents in the 1990s recognized that these agents did not completely abrogate cellular or antibody responses and that more powerful immunosuppression might still alter the course of ALS (34). Moreover, in a relatively recent study of allogeneic hematopoietic stem cell transplantation in ALS, transplantation preceded by only a non-myeloablative conditioning regimen did not benefit ALS patients, despite reported engraftment of stem cells at sites of motor neuron pathology (35). This study provided evidence that stem cell transplantation in the absence of immunoablation may not deliver the greatest potential for success in the ALS patient. In this historical context, the current rush to inject stem cells into the central nervous system without antecedent immunosuppression seems premature because the most definitive trial in immunosuppression for ALS has not yet been performed. Accordingly, a step backward may be required to go forward in the therapeutic fight against ALS.

In this Opinion piece, we propose the use of immunoablation as a therapeutic strategy to abolish the potential neurotoxic effects of the immune response in ALS. It is not our intent to comment on the use of stem cells to renew, repair, or replace damaged motor neurons. In our therapeutic model, reinfused stem cells are required only to replenish or “reboot” the immune system. Without this step, patients undergoing immunoablation will die of opportunistic infections. Hence, immunoablation cannot be performed without subsequent stem cell infusion.

We are aware that there may be concern about submitting ALS patients to immunoablation. However, for almost 20 years, this type of intensive immunosuppression followed by stem cell transplantation has been used with success for the treatment of severe autoimmune diseases (30). Immunoablation and stem cell transplantation is also a standard of care in the treatment of hematological malignancies. In ALS patients, as in patients with autoimmune diseases or hematological malignancies, immunoablation would be expected to be followed by regeneration of a new self-tolerant immune system derived from the reinfused stem cells. The abundance of data suggesting that neuroinflammation contributes to ALS pathogenesis warrants such a trial. The therapeutic concept that in all autoimmune diseases there are intractable cases that subsequently improve with more aggressive immunotherapy also demands the most definitive trial of immunosuppression in ALS. Only then can the autoimmune pathogenesis hypothesis be revived or be laid to eternal rest.

## Author Contributions

All authors listed have made substantial, direct, and intellectual contribution to the work and approved it for publication.

## Conflict of Interest Statement

The authors declare that the research was conducted in the absence of any commercial or financial relationships that could be construed as a potential conflict of interest.
